# Distributed automated manufacturing of pluripotent stem cell products

**DOI:** 10.1007/s00170-019-04516-1

**Published:** 2019-12-04

**Authors:** Maryam Shariatzadeh, Amit Chandra, Samantha L Wilson, Mark J McCall, Lise Morizur, Léa Lesueur, Olivier Chose, Michael M. Gepp, André Schulz, Julia C. Neubauer, Heiko Zimmermann, Elsa Abranches, Jennifer Man, Orla O’Shea, Glyn Stacey, Zoe Hewitt, David J Williams

**Affiliations:** 1 Centre for Biological Engineering, Wolfson School of Mechanical, Electrical and Manufacturing Engineering, Loughborough University, Loughborough, Leicestershire LE11 3TU UK; 2 Present Address: Yposkesi, 26, rue Henri Auguste-Desbruères, 91100 Corbeil-Essonnes, France; 3 CECS/I-STEM, 28, rue Henri Auguste-Desbruères, 91100 Corbeil-Essonnes, France; 4 Fraunhofer Institute for Biomedical Engineering (IBMT), Joseph-von-Fraunhofer-Weg 1, 66280 Sulzbach, Germany; 5 Fraunhofer Project Center for Stem Cell Process Engineering, Neunerplatz 2, 97082 Würzburg, Germany; 6 Present Address: Knappschaft Eye Clinic Sulzbach, An der Klinik 10, 66280 Sulzbach, Germany; 7 Saarland University, 66123 Saarbruecken, Germany; 8 Universidad Católica del Norte, Coquimbo, Chile; 9 NISBC, Blanche Lane, South Mimms, Potters Bar, EN6 3QG UK; 10 Present Address: Oxfordshire, UK; 11 Present Address: Adaptimmune, 60 Jubilee Avenue, Milton Park, Abingdon, Oxfordshire OX14 4RX UK; 12 Centre for Stem Cell Biology (CSCB), University of Sheffield, Western Bank, Sheffield, S10 2TN UK

**Keywords:** Cell therapies, Comparability, Low-grade infection, Mitigation strategies, Monitoring, Process deviation

## Abstract

Establishing how to effectively manufacture cell therapies is an industry-level problem. Decentralised manufacturing is of increasing importance, and its challenges are recognised by healthcare regulators with deviations and comparability issues receiving specific attention from them. This paper is the first to report the deviations and other risks encountered when implementing the expansion of human pluripotent stem cells (hPSCs) in an automated three international site–decentralised manufacturing setting. An experimental demonstrator project expanded a human embryonal carcinoma cell line (2102Ep) at three development sites in France, Germany and the UK using the CompacT SelecT (Sartorius Stedim, Royston, UK) automated cell culture platform. Anticipated variations between sites spanned material input, features of the process itself and production system details including different quality management systems and personnel. Where possible, these were pre-addressed by implementing strategies including standardisation, cell bank mycoplasma testing and specific engineering and process improvements. However, despite such measures, unexpected deviations occurred between sites including software incompatibility and machine/process errors together with uncharacteristic contaminations. Many only became apparent during process proving or during the process run. Further, parameters including growth rate and viability discrepancies could only be determined post-run, preventing ‘live’ corrective measures. The work confirms the critical nature of approaches usually taken in Good Manufacturing Practice (GMP) manufacturing settings and especially emphasises the requirement for monitoring steps to be included within the production system. Real-time process monitoring coupled with carefully structured quality systems is essential for multiple site working including clarity of decision-making roles. Additionally, an over-reliance upon post-process visual microscopic comparisons has major limitations; it is difficult for non-experts to detect deleterious culture changes and such detection is slow.

## Introduction

The purpose of the work reported in this paper was to establish and address the issues associated with automated expansion of human pluripotent stem cell (hPSC), a living material, by partners at three developmental international sites in Germany, France and the UK using a common automation platform. This is an instance of distributed automated aseptic manufacturing of a complex cell therapy. The aim was to run batches for multiple automated passages at each site from a standardised starting material that had been banked at one of the sites. This is significant for a number of reasons: (i) manufacturing of cell therapies for global markets will require aseptic manufacturing in multiple locations in different countries (sometimes within different regulatory regimes); (ii) manufacturing may be close to the point-of-care or use rare (autologous) starting materials [1, 2]; and (iii) the emerging model for international human-induced pluripotent stem cell (hiPSC) haplobanking requires the sharing of starting materials and their subsequent expansion in different countries and within different regulatory regimes [3–5]. The requirement to manufacture products at multiple sites is recognised by both EU and US regulators as challenging for biological products of this complexity [6, 7]. In particular, this requires the demonstration of comparability to ensure that the products are the same after any change. Essentially, in this case, this means that the products at each site and the processes to create them are the same. The practical experimental demonstrator project presented here succeeds a workshop at Trinity Hall, Cambridge, UK [8] held in 2015; the workshop confirmed the context for the present work. It also follows comparability experiments on process changes [9, 10] using the same automated process platform.

Table 1 shows how the current work fits with past work emphasising how achieving comparability within distributed manufacturing has been recognised as a key research gap by the cell therapy community and a challenge by the regulator. The contribution of the work presented here is that it is the first published account of the issues and deviations encountered in attempting to achieve comparability, essentially the production of the same product, at three sites for a biological product representative of a pluripotent stem cell therapy.

**Table 1:** A comparison of the present work with past and other key enabling work to identify the research gap in understanding comparability in the distributed manufacturing of pluripotent stem cell therapies

Therapeutic approach	Cell types	Manufacturing strategy	Regulatory context	Approach	Number of locations	Contribution, significance and research gap	Reference
Clinically led autologous cell therapy	All	Multiple hospital based. See also [11, 12]. For alternatives see [2].	US [6] and EU [7] regulatory environment.	Industry consultation	Many	Identifies the importance of comparability to the roll out of clinically pulled therapies in multiple sites and consequently to the growth of the regenerative medicine industry.	[1]
Generic cell therapies	All	Process scaling and decentralised supply from sites.	US [6] and EU [7] regulatory environment.	Stakeholder workshop	Many	Identification of key research gaps within comparability to be addressed by community.	[8]
Mesenchymal cell therapy	hMSC	Automated process	US [6] and EU [7] regulatory environment.	Experimental demonstrator	One	Measuring comparability of automated and manual process steps	[10, 13–15]
Embryonic stem cell therapy	hESC	Automated process.	US [6] and EU [7] regulatory environment.	Experimental Demonstrator	One	Demonstration of automated hESC culture. Core enabler for work in [16] and current work.	[17]
Haplobanked cell therapy, see [3–5, 18]	IPSC	Automated process (based on [16]; and also see [19] addressing cost).	US [6] and EU [7] regulatory environment.	Experimental demonstrator	One	Measuring comparability of automated and manual process steps.	[9]
Embryonic/Pluripotent cell therapy standard	hPSC	Reference cell lines. See also [20].	US [6] and EU [7] regulatory environment.	Experimental demonstrator	-	Establishment of hPSC reference cell lines. Core enabler for current work.	[21]
Biologics	-	All	US [6] and EU [7] regulatory environment.	Guidelines and overview	-	Approaches to safety [22] and control of contamination [23]. See also [24].	[23]
Pluripotent cell therapy	hPSC	Decentralised automated process emulating multiple hospital or industrial sites.	EU [7] regulatory environment.	Experimental demonstrator	Three international	Practical identification of the comparability issues encountered in manufacturing in multiple sites. Identification of the criticality of agreed approaches to process monitoring and the handling of deviations and the monitoring of low-grade infection.	Reported here

Resolving the differences due to manufacturing at different sites requires that a robust cell culture process protocol and that a common automated processing platform are used at each site. It also assumes the availability and use of a standardised cell line that can be used as a comparator. These are described fully below. With these in place, differences in output are assumed to be a consequence of physical and organisational differences between the sites. Differences in output were anticipated to be an increased statistical variation in process output. However, it was also apparent that other issues would arise during the execution of the demonstrator, but it was not apparent what these issues would be; all project partners understood that a truly informed and practical perspective on the issues encountered would only become apparent after the experiment.

It was anticipated that the work would have three phases: (i) a preparatory phase to identify overall process transfer issues as a consequence of working across international borders within the EU, whereby the transfer takes place from and to regulated development and manufacturing facilities; (ii) a second phase of identifying machine- and protocol-specific issues associated with the expansion and differentiation of cells equivalent to those of therapeutic quality at multiple sites; and (iii) a third phase of routine culture followed by characterisation of the cells to measure the variations from different automated platforms and laboratory locations.

The intent of this experiment was to be a demonstrator emulating the aseptic automated manufacturing of therapies at multiple production sites in order to expose the practical issues encountered in this activity. A key aspiration was the measurement of the additional variation in the product as a consequence of manufacturing at more than one site given that the main automated processing machine to be used was already installed and operating at each site. An ideal experiment would include three production facilities following the same quality management system (QMS) across the three sites. However, it is important to recognise that several pragmatic decisions were made to facilitate the experiment as shown in Table 2. The largest of these is that the three facilities did not operate to GMP conditions but were considered to be close to it because of their translational development orientation. The risks arising from these decisions are shown in Table 3.

**Table 2:** Pragmatic decisions made during the design of a three-site experiment

No	Ideal scenario	Replacement	Justification
1	Use the production process for a pluripotent stem cell derived therapy to transfer across to three production facilities.	Banking of pluripotent stem cell-like-cells (the surrogate product) used as the production process in this experiment. The cell line chosen is the embryonal carcinoma cell line EC 2102Ep [21].	Both cells and production protocols need to be publicly or commercially available with permissions to transfer across multiple sites. In this experiment, the chosen cell line is commercially available, and the protocols derived are not commercially sensitive, hence they can be transferred across the three sites. Embryonal carcinoma cell lines test positive for a panel of gene expression markers common with pluripotent stem cell lines. The 2102Ep cell line is permitted for use in all the countries where this experiment is being carried out, in contrast to human embryonic stem cells (hESCs) which are restricted for use in many countries [25]
2	Use three production facilities with a GMP certificate for production of hPSC-derived cell therapies	Three development facilities, which are currently working towards industrialisation of cell therapy products. The deviations in the non-GMP setting highlight and confirm areas of risk.	GMP-qualified labs are not practically available for such an experiment, as there is currently insufficient GMP capacity [26], and they have very high-associated costs [11]. The use of a non-GMP setting in development facilities should provide sufficient sensitivity to detect failure. A gap analysis based on EU Regulations [7] and WHO Biology Laboratory Guidelines [22] was performed (Table 3) to demonstrate the differences anticipated and observed by the site teams.
3	Use of a production process for a mature therapy where the production process has been developed sufficiently to reflect its mature status.	The experiment uses an embryonal carcinoma cell line as a surrogate product and a production protocol automated on the Compact SelecT (Sartorius Stedim, Royston, UK) which is a development tool for expansion of adherent cell cultures.	Mature production processes are product specific. For pluripotent stem cell-derived products production processes are currently limited or non-existent. Use of product surrogates allows for identification of critical process parameters. Automation is essential in any mature production pipeline.
4	In-process quality control is automated, and the batch records are maintained by the automated platform.	Not all the Compact SelecT platforms utilised at the three sites had integrated microscopes for the in-process visual verification of the culture progress. In this case, the operators at the sites are to manually view the flasks.	A verification of the progress of the production process at each step is not a regulatory requirement for a qualified process. During this experimental process, operators will manually view the flasks and take images. These images are to be shared with the experimental teams at the three sites. Well maintained laboratory notes are to act as the batch record.

**Table 3:** Risks identified during the experimental design

Ideal scenario (GMP production)	Pragmatic scenario (development laboratory)	Risk
Facility		
The design and construction of the facility is based on its suitability to the production environment where the needs of the product are paramount. There is usually separation of areas to minimise mix-ups/contamination and careful attention to process flows. There are prescriptive regulations regarding lighting, plumbing, sewage and washing facilities under GMP, as well as specific product quality assessment tests and acceptance criteria at different production stages.	Design and construction of the research and development facilities are normally prescribed by health and safety concerns for the users. Segregation might be required for preventing mix-ups or contamination, but it is not obligatory. Other features of the facility including construction etc. are not universally defined. These decisions are set locally so are often not comparable across sites.	Potential increased risk of process deviation (for example due to facility temperature variances) and increased risk of product contamination and failure to pass acceptance criteria.
Production team		
Responsibilities of all the operators and supervisors should be in written procedures. The production team and supervisors will be fully trained in their respective roles.	The training in the tasks will be based on the experiments to be performed.	Increased risk of deviations to the protocols.
Training assessed and recorded formally and regularly	Critical assessment made of the protocols of each laboratory.	
Release of product performed by a Qualified Person (QP) leading a team of quality assurance and quality control professionals.	There is often no product other than the results from testing and they are released by the supervisor of the team.	
Equipment and consumables		
Equipment for use in production and testing must be qualified for the use. Data generating equipment for product testing must be calibrated regularly. The accuracy, sensitivity, specificity and reproducibility of test methods should be established and documented.	Equipment must be appropriately, maintained and calibrated. Equipment qualification with formal record keeping is not obligatory.	Increased risk of incorrect read-outs.
Automation platform		
Automation platform qualified for the unit operation in the site. The site provides the required environment for the automation platform.	Automation platform for the unit operation installed correctly.	Increased risk of protocols varying across sites
Procedures		
All SOPs are drafted based on guidelines by qualified personnel and approved by the quality control (QC) unit of the production facility.	SOPs are based on the local requirements and the equipment manuals. They are written and approved by research and development staff rather than quality or regulatory professionals.	Increased risk of protocols varying across sites. Increased risk of product variability.
SOPs are standard for the activities in the production facility. Each production task will be recorded in batch records following the SOPs for the production facility.	It is possible that different experiments have ‘experimental operating procedures’ which are specific to the experiment and may override SOPs for the facility.	
All batch records are signed off by the operating personnel along with the personnel verifying the steps (dual control of procedures/records). These are maintained long term.	Sign-off for experimental records is not a formal process. Often operator sign-off is sufficient.	
Testing is prescriptive and will be performed in the same manner for all batches.	Testing is based on the requirements of the experiment.	

Overall, analysis of the results obtained highlighted the significance of unexpected differences and deviations during the second phase of the project. This included the challenges in handling low-grade contamination, whilst highlighting the crucial importance of managing this. As the risks identified in Table 3 show, this is most likely a reflection of the settings and gaps in the preparation and execution of the experiment. This is a consequence of the availability of resources including people and time, and of the difficulty of imposing a consistent quality system and close to GMP-like approach on three different development organisations in the absence of commercial drivers.

This publication consequently reports on the results of an experiment to measure and record deviations (both anticipated and unanticipated) when the mature manufacturing protocol of a pluripotent stem cell–derived therapeutic is transferred across multiple development sites. The paper begins with a presentation of the materials and methods used, the key results of the culture process followed by a description of the deviations encountered. It then continues with a discussion of the results obtained, including the deviations encountered and their consequences. It concludes with a summary of the learning from the experiment.

## Materials and methods

The production protocol was performed at three international developmental sites. These were the Centre for Biological Engineering, Loughborough University (UK); the Fraunhofer Institute for Biological Engineering (Germany) and Institute for Stem cell Therapy and Exploration of Monogenic diseases (I-Stem, France). The developmental sites are here referred to as sites, 1, 2 and 3; these numbers have been randomly assigned. The quality assessment was performed at NIBSC (UK).

### Cell lines

This work has been executed using the embryonal carcinoma (EC) 2102Ep cell line (2102Ep, GlobalStem, USA) derived from primary human testicular teratocarcinoma [21]. The cells were expanded in-house to make a master cell bank, followed by corresponding working cell banks. All procedures performed were in accordance with the ethical standards of the institutional and/or national research committee and with the 1964 Helsinki declaration and its later amendments or comparable ethical standards. EC lines are robust cell lines that allow for comparison studies between laboratories since they grow without the need for feeder cells, are relatively simple to passage and resist spontaneous differentiation and are a rich source of the proteins and mRNAs used to characterise hPSCs [21]. A National Institute for Biological Standards and Control (NIBSC) hiPSC line, NISBC8, a quality-controlled bank of 2102Ep embryonal carcinoma cells, the F9 embryo teratocarcinoma cell line and a UK Stem Cell Bank (UKSCB) NIBSC internal standard n210EP-UKSCB were used as flow cytometry control lines.

### Manual cell culture protocol

Unless otherwise stated, all reagents and consumables used were purchased from Fisher Scientific, Leicestershire, UK. EC 2102Ep were expanded at high density on T175-cm^2^ tissue culture plastic flasks (Corning Lifesciences, NY, USA) at 37 °C in a humidified chamber containing 5% CO_2_ in air. The growth medium used was Gibco DMEM high glucose with glutamax supplemented with 10% (*v*/*v*) foetal bovine serum, qualified and heat-inactivated (FBS). The growth medium was changed every 48 h.

Cells were passaged every 3 days; the spent medium was aspirated, and the cells were washed with PBS (−Ca^2+^/−Mg^2+^), 250 μl/cm^2^. The cells were detached using 7 ml 1xTrypsin-EDTA (0.25%, *v*/*v)* phenol red solution for 5 min at 37 °C. Cells were passaged every 3 days when flasks were heavily confluent and split according to cell count and the appropriate required cell density. Wash medium was added at twice the volume of Trypsin-EDTA used and the cell suspension was centrifuged for 5 min at 300×*g*.

### Cell counting

The NC-3000™–automated image cytometer (ChemoMetec, Denmark) was used to perform cell counts. Cell counts and viability were measured using the cell viability and cell count assay following the manufacturer’s instructions. The assay utilises a commercially available pre-mix of acridine orange base N, N, N′, N′-tetramethylacridine-3,6-diamine monohydrochloride (AO) uptake and 4′,6-diamidino-2-phenylindole, dilactate (DAPI) exclusion (Solution 13, eChmoMetec, Denmark). Solution 13 was mixed into the cell suspension at a ratio of 1:19; prior to loading into a NC-Slide-A8 (eChmoMetec, Denmark).

### Cell banking and cryopreservation

An EC 2102Ep master cell bank was created in-house; all cells were banked at Passage 46 (P46) and a total of 353 ml cryovials each containing 1.5 × 10^7^ cells were suspended in CryoStor solution according to the manufacturers protocol (Sigma-Aldrich, UK). The vials were stored in a Mr Frosty CoolCell® passive cooling device for 24 h at − 80 °C, before being transferred to the vapour phase of liquid nitrogen where they were stored prior to experimentation.

### Setup of comparability experiment

The team from site 1 coordinated the experiment as well as being the first site for the experiment. The experiment coordination package included producing the 35-vial-sized working bank for the experiment which was distributed under controlled shipping conditions to the other two sites along with essential consumables including media, pipettes and tubing; sharing a common operating procedure for the CompacT SelecT; and training on the CompacT SelecT.

### Automated cell culture

#### Machine

The TAP Biosystems’ CompacT SelecT–automated cell culture platform (Sartorius Stedim, Royston, UK), which utilises an incubator carousel to store cell culture flasks, multiple peristaltic pumps to dispense cell culture reagents and a robotic arm to replicate many of the process steps involved in manual cell culture, was utilised in this study. This platform also incorporated a Cedex analyser–automated cell counter (Roche, Switzerland); the system utilises Trypan blue exclusion and automated imaging software to determine viable cell density, viability and aggregate rate. The CompacT SelecT has previously been demonstrated for the culture of hMSCs [13, 14, 27], hESCs [17] and hiPSCs [10, 16] as aggregates.

#### Machine preparation and calibration

Prior to the performance of any automated protocol on the TAP Biosystems’ CompacT SelecT platform, the machine was prepared for use by ensuring a sufficient number of pipette tips were loaded, sufficient new T175-cm^2^ flasks were available, that adequate volume of reagents were loaded aseptically and that the required sterile plastic tubing (Watson-Marlow Pumps, Falmouth, UK) was connected to allow for reagents to be pumped using the peristaltic pump system. To ensure that the required volumes of reagent are dispensed throughout each protocol, a calibration step is performed prior to each CompacT SelecT protocol. Briefly, the plastic tubing was primed, and a small volume of reagent was dispensed into a BD Falcon TMT175-cm^2^ tissue culture flask (BD Bio-sciences, San Jose, USA). The flask was then ‘borrowed’, and the contents were weighed on digital scales. ‘Borrowed’ refers to the programming terminology whereby the machine is instructed by the operator to eject the flask from the machine. This step allows the operator to determine the volume of reagent dispensed, assuming that 1 mL of reagent weighs 1 g. This value can then be entered into the CompacT SelecT software in order to calibrate the peristaltic pump system, which adjusts the subsequent dispensing steps accordingly.

#### Dry run and automated culture emulation

The CompacT SelecT–automated protocol consisted of the following: (i) dry run—a software compatibility run with each of 4 sub-protocols including media change, passaging the cells split 1:3 and 1:1 and sampling (but without cell culture media). (ii) Wet run—using PBS/ddH_2_O to emulate culture media, with each of four sub-protocols after repair service and preventative maintenance (PM).

#### Automated cell culture protocol

**Fig. 1:**
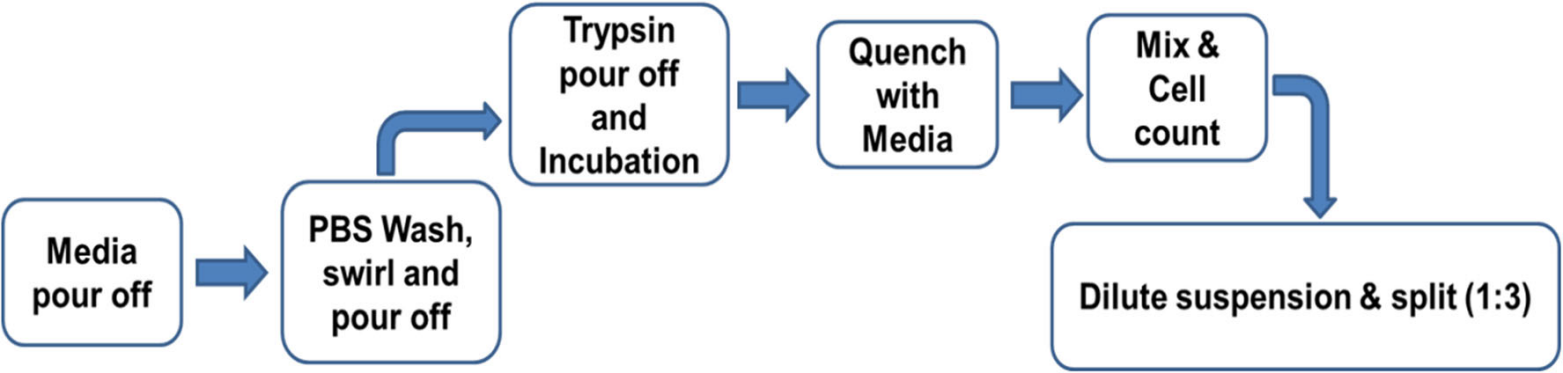
Schematic summary of the automated cell culture protocol on the CompacT SelecT. The robotic arm pours off the media prior to a washing step in PBS to remove any residual media. The trypsin pour-off step is performed so that only a minimal coating of the chelating agent is in contact with the cells since the automated system is incapable of centrifugation. This is followed by quenching with FBS enriched media whereby the proteins in the FBS neutralise the trypsin. The cells are mixed, and a cell count is performed using the Cedex automated system. An appropriate volume of media is then added to the new flasks to obtain the desired seeding density

**Fig. 2:**
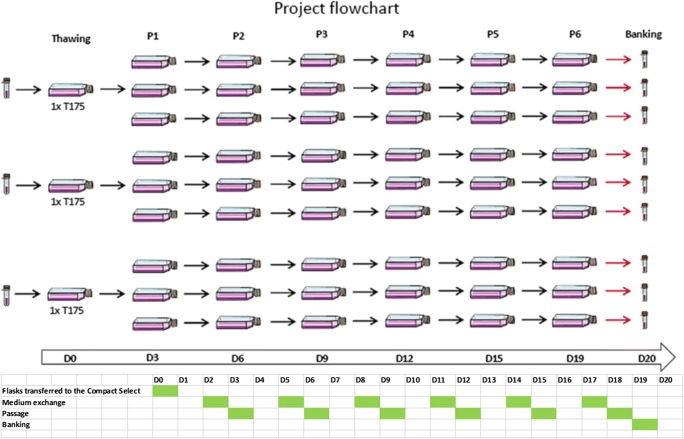
Schematic representation of the EC 2102Ep expansion protocol, demonstrating the three cryovials that were thawed and initially seeded into three corresponding T 175 cm^2^ prior to expansion in triplicate over six passages

Three cryovials of 2102Ep cells were thawed and cells from each vial were transferred directly into a T175-cm^2^ flask. T175-cm^2^ flasks were loaded into the CompacT and incubated in the CompacT incubator at 37 °C with 5% CO_2_. Media exchange was performed 48 h post-seeding and cells were sub-cultured the following day (day 3) into 3 daughter T175 cm^2^ flasks according to cell count, so that each daughter flask contained 1.5 × 10^7^ cells, equivalent to 85,000 cells per cm^2^ (Fig. 1). Cells were sub-cultured for six passages (Fig. 2). Cell samples were collected for flow cytometry analysis after each passage and stored at – 80 °C.

#### Culture protocol optimisation/repeatability improvement

An automated protocol was first derived for pluripotent stem cells expansion by observation of manual culture by Loughborough University engineers of expert Cambridge University biologists [16] working with a well-defined hiPSC line. Parameters for this protocol were then adapted to the expansion of the 2102Ep cells [19]. The initial protocol was based on 1:3 or 1:4 split ratios for cell passage. The protocol was revised and improved, and a cell count step was added whilst performing the splits to both manual and CompacT automated protocols. The CompacT improved protocol included increased shaking time and pipette mixing with elimination of sample pooling steps since the machine does not use centrifugation and permit flask-by-flask sampling.

### Immunophenotyping using flow cytometry

Immunophenotyping using flow cytometry (BD FACSCanto II, San Jose, CA) was performed on samples from site 1. The complete analysis was performed by NIBSC to remove any potential bias in the interpretation of results. Excess cell suspension was removed from cells at passage 1-5. The cells were centrifuged at 300×*g* for 5 min prior to suspension in CryoStor freezing solution and storage at – 80 °C. Since the detached cells from passage 6 were at the end of the experimental procedure, all cells were frozen down and stored, as opposed to just the excess cells. Prior to staining the cells were thawed and pelleted via centrifugation for 5 min at 300×*g*, room temperature, washed in 1xPBS, and then fixed in Cytofix/Cytoperm solution (BD Biosciences, UK) at 4 °C for 20 min.

Fixed cells were washed with 1xPBS and assessed for the stem cell markers, Oct-4, Nanog, SSEA4, SSEA1 and TRA-1-60, using specific antibodies (R&D Systems Cat: 560477, 506122 and 561300). Briefly, cells were permeabilised with BD perm/wash buffer for 10 min at 4 °C. The antibodies (concentrations according to manufacturer’s instructions) were incubated for 20 min at 4 °C and were protected from light. The cells were then washed in A perm/wash buffer (×3) and run on the BD Canto II flow cytometer according to manufacturer’s instructions.

### Robust sample handling and logistics

Frozen 2102Ep cryovials were transferred between sites via courier and on dry ice in a polystyrene container. Sampling was performed after each passage step manually and the remaining cell suspension of each flask were added to CryoStor solution and were frozen at – 80 °C.

## Results

As has been described in “Section 1”, this experiment is a demonstrator where it was anticipated that practical issues would be encountered. It is therefore challenging to present the results in a conventional serial manner. Consequently, we present them structured as those arising in the preparatory phase, biological variations and other key subsequent themes that arose during culture, as below.

### Preparatory phase

The organising site team visited the three sites prior to starting the experiment. Several differences and deviations were identified between the three manufacturing sites prior to, and during the execution of the automated expansion of the 2102Ep cell line. These variations were mainly linked to the processing machine; these included differences in air flow, incubator temperature and software compatibility. Furthermore, organisational issues were also apparent (Table 4). To elaborate, (i) laminar and negative air flow deviations occurred, although these were minimal and remained within specification, thus the consequences were negligible. (ii) The incubator CO_2_ % at site 2 was reading unusually high (26%) despite servicing being carried out; hence, an alternative (iii) incubator was used to minimise any detrimental consequences on the culture. Incubator temperature was 0.6 °C higher at site 3, although the effect to the 2102Ep line would be minimal due to the robust nature of the cells. (iv) The integrated microscopy system was absent in the automated system used at site 1 due to it being a first-generation machine, as a result, visual monitoring of cell phenotype was therefore omitted from the protocol. It is possible that if the addition of a visual monitoring step had been included in the SOP, this step would have detected the contamination that was observed at site 3. (v) Software incompatibility issues manifested themselves as the inability of the three machines to use the same software protocol across the three sites. Consequently, at site 3, the protocol required some adaptation.

**Table 4:** Summary of major differences and deviations identified between sites prior to and during expansion of EC 2102 Ep cells

**Specification**	**Site 1**	**Site 2**	**Site 3**
Air flow			
Laminar flow: 0.35–0.55 (m/s) Negative flow: 0.4–0.7(m/s)	Laminar flow: 0.435 Negative flow: 0.647	Laminar flow: 0.454 Negative flow: 0.504	Laminar flow: 0.481 Negative flow: 0.576
Incubator CO_2_ %	5%	26%*	5.5%
Incubator temperature	37 °C	37 °C	37.6 °C
Lab based SOPs for machine decontamination/cleaning	Two-stage cleaning, disinfectant followed by rinsing and manual wiping	Vapour hydrogen peroxide decontamination	In-house vapour hydrogen peroxide decontamination
Organisational issues	First machine	Required machine move and use of alternative incubator	Prohibition of weekend working

*The sensors were faulty/not calibrated at site 2, resulting in such a high CO_2_ read-out

Other differences included those in the quality systems of the sites. The three sites had been chosen because they all work on pre-production development activities and have a laboratory-based quality system that has been developed to include a demonstration of the reliability of results generated, and importantly that reflected the quality systems of associated production facilities. However, several differences were identified during the preparatory phase. A significant example of the differences between sites were the standard operating procedures (SOPs) for regular cleaning. For instance, at site 1, the SOPs were developed based on the understanding that a two-stage cleaning with manual wiping (first with the disinfectant followed by a rinse) of every surface was the best method for regular cleaning. In contrast, at site 3, the SOPs reflected the procedures in the production facility where frequent vapour hydrogen peroxide decontamination was sufficient. In both cases, the effectiveness of the respective regular cleaning was validated for the associated production facility. However, this validation was not conducted in the development laboratories; hence, the two quality systems could not be directly compared. This difference in the cleaning procedures particularly affects the regular cleaning of equipment such as biological safety cabinets, incubators and the automated processing machine. Consequently, it was difficult to pinpoint the exact causes of contamination.

Other instances of differences in the quality systems encountered included the following: different maintenance regimes for essential equipment including biological safety cabinets, incubators, autoclaves; sharing or otherwise of pipettes; standards for training of new staff; and routine testing for contamination such as mycoplasma.

### Biological variations across sites

#### Cell culture

The cells grew as a uniform monolayer with a high nucleus-to-cytoplasm ratio and prominent nucleoli [21]. 2102Ep cells were attached to untreated tissue culture plastic flasks and were maintained at high density. Cells were passaged every 3–4 days according to cell count and confluency. Cells maintained this morphology following several manual passages; cell clusters were also observed from the onset of the manual expansion, as the cells became increasingly confluent, although these observations were not recorded.

#### Cell numbers and viability

**Fig. 3:**
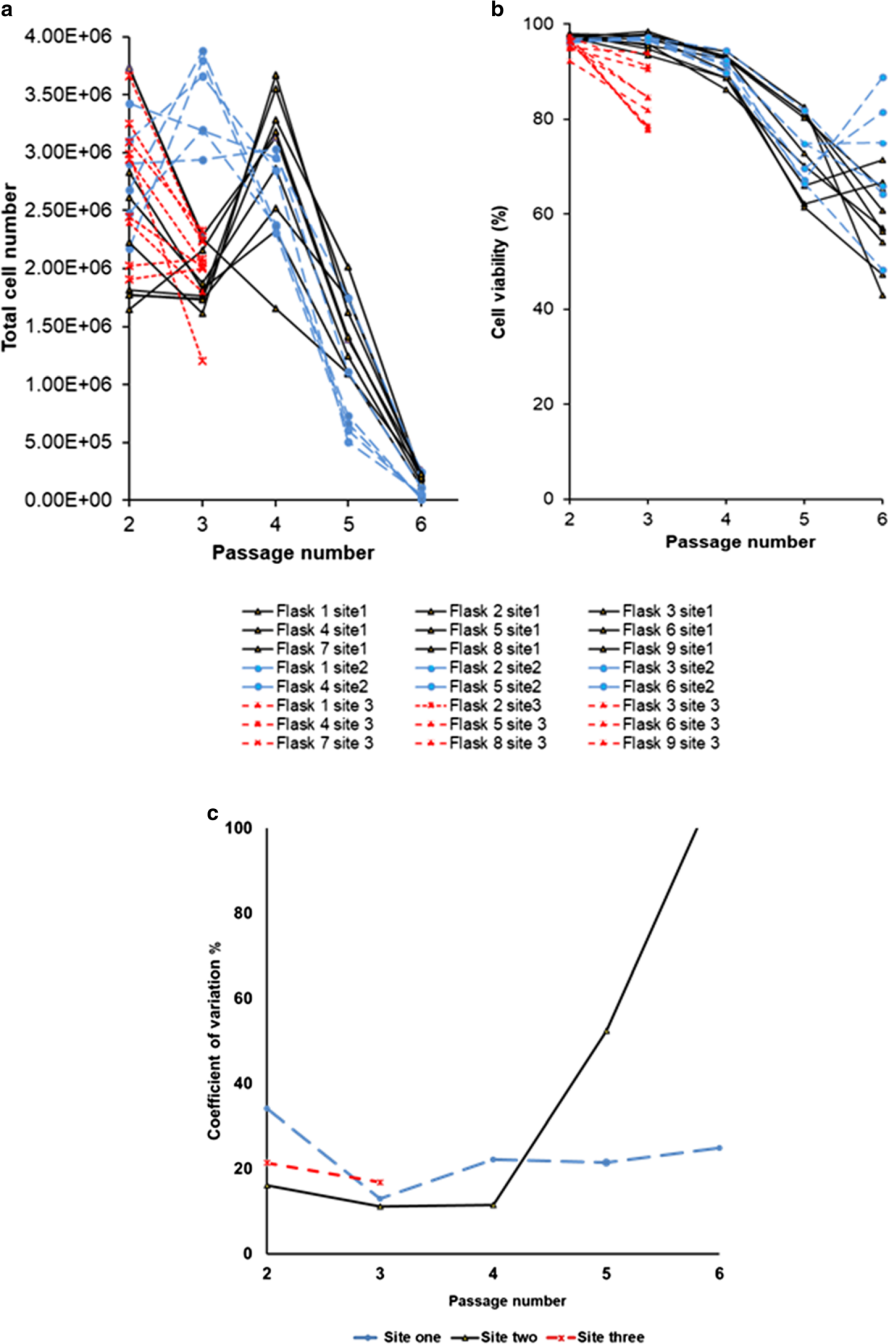
**a** Total number of cells per flask based on Cedex automated cell counting (left axis) at different culture passage; variations in flask numbers are the result of having to exclude one flask at passage 1 at site 2 due to insufficient cell recovery post thawing. **b** Comparison of flask-to-flask cell viability when expanded at multiple manufacturing sites. **c** Percentage of co-efficient of variation (CV) for the total cell number; black solid line represents site 1, blue dashed line represents site 2, and red small dashed line represents site 3

The result of the Cedex–automated cell count demonstrated a flask-to-flask variation in the total cell number following the first passage at all sites; this was despite the identical initial cell seeding density (Fig. 3). The total number of cells at site 1 initially decreased to a combined average of 1.92 × 10^6^ cell/ml at passage 3 (*n* = 9, SD = ± 0.25 × 10^6^ cell/ml), prior to increasing to 2.92 × 10^6^ cell/ml at passage 4 (*n* = 6, SD = ± 0.64 × 10^6^ cell/ml). Despite this spike in cell number, from passage 5 onwards, there was a significant decrease in the total cell number recorded, with less than 0.5 × 10^6^ cell/ml at passage 6. It is postulated that the drop in cell number at passage 3 was due to the initial change to an automated culture; the subsequent increase in cell number at passage 4 is potentially due to the cells acclimatising to the change in culture processing, in particular the requirement for additional pipetting in the automated protocols to reduce cell clumping.

At site 2, the initial increase in cell number to 3.45 × 10^6^ cell/ml at passage 2 (*n* = 9, SD = ± 0.38 × 10^6^ cell/ml) was followed by a significant decrease in total cell number at passage 4; this continued at both passages 5 and 6, whereby less than 0.5 × 10^6^ cells were counted respectively. As speculated above, this may be an inherent artefact of the cells adapting from manual to automated cultures.

Cell viability reduced substantially following passage 4 to approximately 80% at both sites 1 and 2 (Fig. 3B). Flask-to-flask variability was low at both passages 3 and 4 for both sites 1 and 2; deviations between flasks only began to present following passage 5. The trend in reducing cell viability with increasing passages continued for the majority of the flasks following passages 5 and 6 at both sites 1 and 2. In addition, flask-to-flask variation increased at both sites; in particular at site 2, whereby viability ranged from 66.4 to 81.9% at passage 5 and 48.3 to 88.9% at passage 6. An increase in cell viability at passage 6 was observed in 3 of the flasks expanded at site 2 only.

At site 3, the experiment was terminated at passage 3 due to higher observed deviations in cell viability data and lower cell growth, most likely as a result of cell culture contamination. Specifically, cell viability at site 3 were visibly lower following passage 3 with greater flask-to-flask variation, between 77.7 and 93.2% recorded.

In addition, the flask-to-flask variation in cell count increased at all three manufacturing sites from passage 2 onward. The variations were more apparent in the earlier passages. All experimentation at site 3 ceased at passage 3 due to detection of cell culture contamination. The co-efficient of variation (CV) data demonstrated that there are significant differences in process outcome regarding the cell numbers despite using the same process, machine and protocol across and within sites (Fig. 3C). This is a representative of an accumulation of the different sources of variation, discussed at length in the following sections.

### Cell phenotyping by flow cytometry

Flow cytometry was only performed on cells cultured at site 1; the cells were unfortunately lost due to a courier error at site 2 and were terminated in the experiments performed at site 3. Flow cytometry revealed a variation in the expression level of all four pluripotency markers analysed when comparing 2102Ep cells at earlier and later passages.

**Fig. 4:**
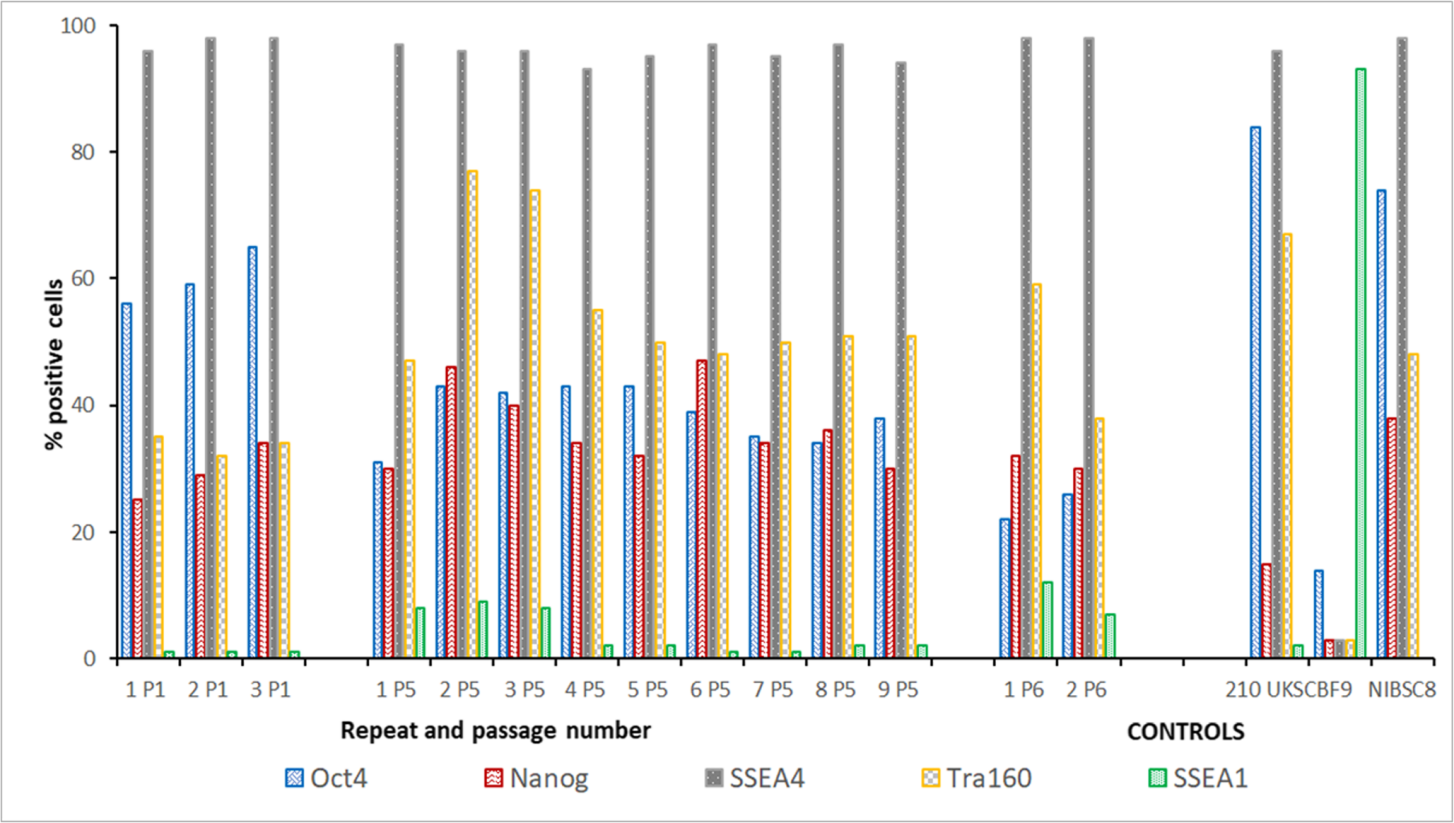
Flow cytometry analysis performed on cells cultured at site 1 based on the EC 2102Ep population positive percentage expression level of pluripotency markers

SSEA-4 expression levels remained stable at approximately 97% when comparing flask-to-flask and passage numbers (Fig. 4). The results were also comparable with the NIBSC internal standard 2102Ep cells and the hiPSC line NIBSC8. The TRA-1-60 population positive percentage showed lower levels of expression in the in-house 2102Ep cells when compared with the n210EP-UKSCB internal standard sample but was higher than the NIBSC8 control with average expression of approximately 54.7%; in comparison with 67% (n210EP-UKSCB internal standard) and 48% (NIBSC8) expression respectively.

The result of Oct3/4 staining exhibited greater flask-to-flask variation, and increased variation between earlier and later passages in comparison with the NIBSC standard samples (Fig. 4). The average expression level was initially 59% at passage 1 and subsequently 24% following the sixth passage. This potentially correlates with the reduced cell viability, although this could also be due to the cells starting to differentiate. A definitive reason for this could not be determined since only pluripotency markers were tested. Further experimentation would be required to rule out the possibility of differentiation occurring. Oct 3/4 levels in the NIBSC8 samples were much higher at 77% on average.

The transcription factor Nanog positive population percentage matched with the NIBSC8 samples (35% on average and below 40% respectively) whilst the n210EP-UKSCB sample expression level was reported much lower at approximately 10%. Overall, Nanog expression levels were reported as relatively stable between different flasks and passage numbers. The Nanog positive percentage expression was approximately 22% in flask 3 at passage 5, with the highest expression level (47%) reported in flask 6 at passage 5.

N.B. All samples were subsequently destroyed, some due to the lack of management oversight associated with academic development settings.

## Discussion

### Cell line choice

Ideally, a clinically relevant pluripotent stem cell line, either hESC or hiPSC, adapted to automated expansion, would have been used for the comparability experiment. However, due to restrictions in Germany and France on the use of hESC, it was necessary to use an alternative. Comparator cell line options discussed included the Cellartis (now part of Takara Bio Europe) SA001 hESC line. This cell line has been used to derive keratinocytes stored in the cell banks at Passage 62, and these cells had been adapted by I-Stem (one of the project partners) to automate the expansion and differentiation to epidermis protocols. Interactions with the wider community working on pluripotent stem cells also occurred at the Stockholm meeting of the Global Alliance for hiPSC Therapies (GAiT) [18] in June 2015; however, the delegates were not able to suggest an available iPSC line of clinical relevance at that time.

The UK Stem Cell Bank (UKSCB), part of the UK National Institute for Biological Standards and Control (NIBSC, [20]), was represented at GAiT [18]. The NIBSC have a mission for developing biological reference materials. Researchers at the UKSCB had been working to develop the EC 2102Ep line as a reference material (i.e. a ‘ruler line’) to be used for developing and qualifying processes and equipment. Whilst close to being thought of as an emulation of an embryonal stem cell line, it is not considered to have the sensitivity to culture conditions associated with these and therefore cannot be viewed as a comprehensive standard. It has been previously characterised by Josephson et al. (2007) [21]. EC 2102Ep was consequently chosen as the best available reference line.

### Cell count and viability

Cell count and viability varied from flask to flask and from site to site. Apart from the previously mentioned deviations and differences in the manufacturing sites and the CompacT SelecT machine specification, there are other possible explanations for cell count variation. One possible explanation is that errors in cell count occurred due to the high percentage of cell aggregate formation. The selection of dissociation agents and an optimisation of cell detachment duration is required to minimise the formation of cell aggregates. Another reason for an error in cell number could be due machine capability limitations and the number and duration of pipette mixing steps. Although the number of pipette mixing steps and mixing duration were adjusted, and optimised, numerous cell aggregates were still visible following the mixing step. Furthermore, the CompacT SelecT default pipette size is 10 ml, which is not suitable for breaking down the cell aggregates into single cells; therefore, a change in manufacturing design of pipette holder is needed for smaller liquid handling volumes, potentially to include the use of 2-ml pipettes. In addition, Cedex the automated cell count device could be a possible source of error. Cedex utilises the Trypan blue reagent for count and viability, by introducing a parallel cell count using more advanced cell counting devices such as the NucleoCounter NC-3000™. This would reduce the potential error, whilst generating more comparable and accurate cell counts and viability recordings. The initial flask-to-flask variation in cell count that occurred at all sites suggested differences in recovery from cryopreservation and/or during the manual thawing process.

### Cell phenotyping by flow cytometry

A comparison of the phenotype data from site 1 and NIBSC (control data) suggested that the variant of the EC 2102Ep may not be sufficiently stable for the use as a standard cell line. This was evidenced by variation and discrepancies in the expression of pluripotency markers (Fig. 4). These challenges have previously been recognised and reported [21]. This could be further exacerbated by the automated culture protocol due to the removal of the centrifugation step. The expression of the pluripotency markers SSEA-4, TRA-1-60, Oct3/4 and Nanog did not remain sufficiently stable from passage-to-passage and flask-to-flask variations were observed. However, such changes may also be a consequence of the low cell viability/poor growth rate rather than the stability of the cell line.

### Relationship between total cell count and viability

The cell count and viability analyses revealed considerable variability across three sites and between passages. Although the experiment had to be terminated at passage 3 at site 3, the reduction in total cell count coincided with the reduction in cell viability that occurred between passages 2 and 3. Despite the spike in total cell count that was recorded at passage 4 at site 1, from passage 5 onwards both total cell count and viability decreased. It was observed that the decline in cell viability that occurred between passages 4 and 5 at site 2 also coincided with a reduction in cell count. However, three of the six flasks showed an increase in cell viability at passage 6, despite the total cell count declining to less than 0.5 × 10^6^ cells/ml.

### Deviations

This experiment has allowed for the identification of deviations, the types of potential deviations and an assessment of their impact. This demonstrates that the experiment was more representative of preparatory engineering runs for operational qualification (OQ), process qualification (PQ) and validation purposes. The third phase of our experiment was anticipated to allow the comparison of the output of stable production runs. However, we were not able to progress the experiment fully through the envisaged third phase because of deviations. The deviations observed in the experiment are discussed in detail below.

#### Deviations in manufacturing and deviations in regulated manufacturing

It is appropriate to consider process deviation in a conventional manufacturing context. People, machines and systems execute process protocols step by step; even if the individual steps are complex within themselves, these steps take the process and product from state to state. Some deviations may be consequences of installation differences. A process deviation occurs when the state achieved at the next step is not the desired state. Process deviations can be of different types. Some process deviations are fatal and it is clear that the product must be scrapped. In other circumstances, it may be possible to repair the product by a subsequent rework process. However, it is also possible in some cases to recover the process or product within the individual machine or system. Recovery returns the system to the desired state either at the same or next step/s using conditional contingency actions that depend on the actual system state.

Informal discussions within the UK as the experiment progressed amplified the regulatory perspective particularly with respect to actions that can be taken after a deviation in multiple site settings. These were predicated by the assumptions that each site had equivalent equipment qualified in the same way, that starting and raw materials were qualified, and that the product produced must not breach pre-set specifications within clinical trial or marketing authorisation. This discussion emphasised that the level of discretion permitted to the qualified person (QP) in the handling of deviations is currently controversial and open to interpretation, with the need for significant training on individual sites, and that the handling of unexpected deviations was complex and difficult. The need for decisions to be made quickly and sometimes remotely poses further challenges. The need for increased vigilance to monitor for deviations once known was emphasised in this demonstrator experiment. This highlighted the requirements for thorough failure mode and effect analysis (FMEA) to be performed in the process design.

Should this approach be taken such actions must necessarily be within the defined path in the clinical trial or marketing authorisation.

#### Anticipated and unexpected outcomes

**Fig. 5:**
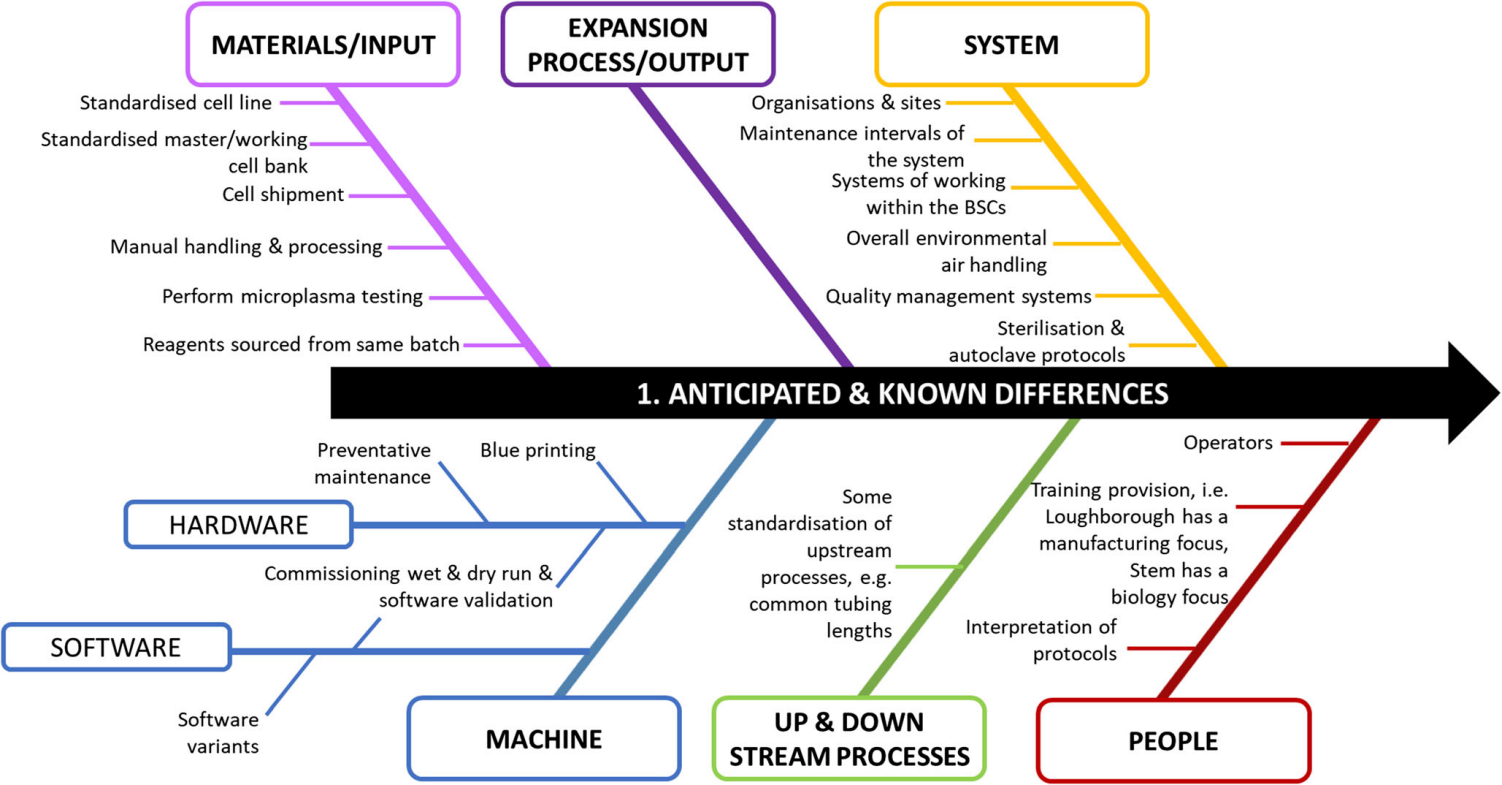
Ishikawa or fish-bone diagram illustrating the anticipated and known manufacturing process deviations identified at the start of the experiment grouped into broad areas and by individual issues

As previously described (Table 4), several differences between sites were initially identified and several key actions were initiated to minimise the variance between sites presented here as an Ishikawa or fish-bone diagram (Fig. 5). These actions were typically those that would have been managed by the installation, commissioning and qualification, training, maintenance and preventative maintenance (PM) processes. This included machine repair and the requirement for subsequent overall equipment effectiveness (OEE) growth before the equivalent of process qualification (PQ). This required considerable interaction with the supplier, with some delays resulting from the lack of engineering resource. Overall, the implemented actions were able to set the machines at the different sites into the desired state, which was confirmed by a ‘wet run’. The experiment demonstrated that there was little clarity of specification and the range of settings across the sites gave reduced achievable range. Consequently, this resulted in a tighter process specification across the sites. This drifting increase of constraints is known by some as the ‘specification trap’. Further compromises between sites were also required due to differences in quality systems, this included approaches to asepsis, different work patterns and engineering changes due to differences in site layout. For instance, site layouts meant that there were differences in the length of feed tubing. An agreement on a common agreed core process protocol was also necessary.

A protocol was initially written at site 1 and distributed to all sites for the CompacT SelecT automated process; any deviations were identified, and local specific changes were included and approved, i.e. the inclusion of an inspection step; ensuring that the process was equivalent at the three sites. However, insufficient attention was paid to gathering and compiling the expertise of operators at all sites into the final agreed common protocol. This emphasised the requirement for an inclusive responsibility for the writing of all SOPs as part of the process transfer process.

**Fig. 6:**
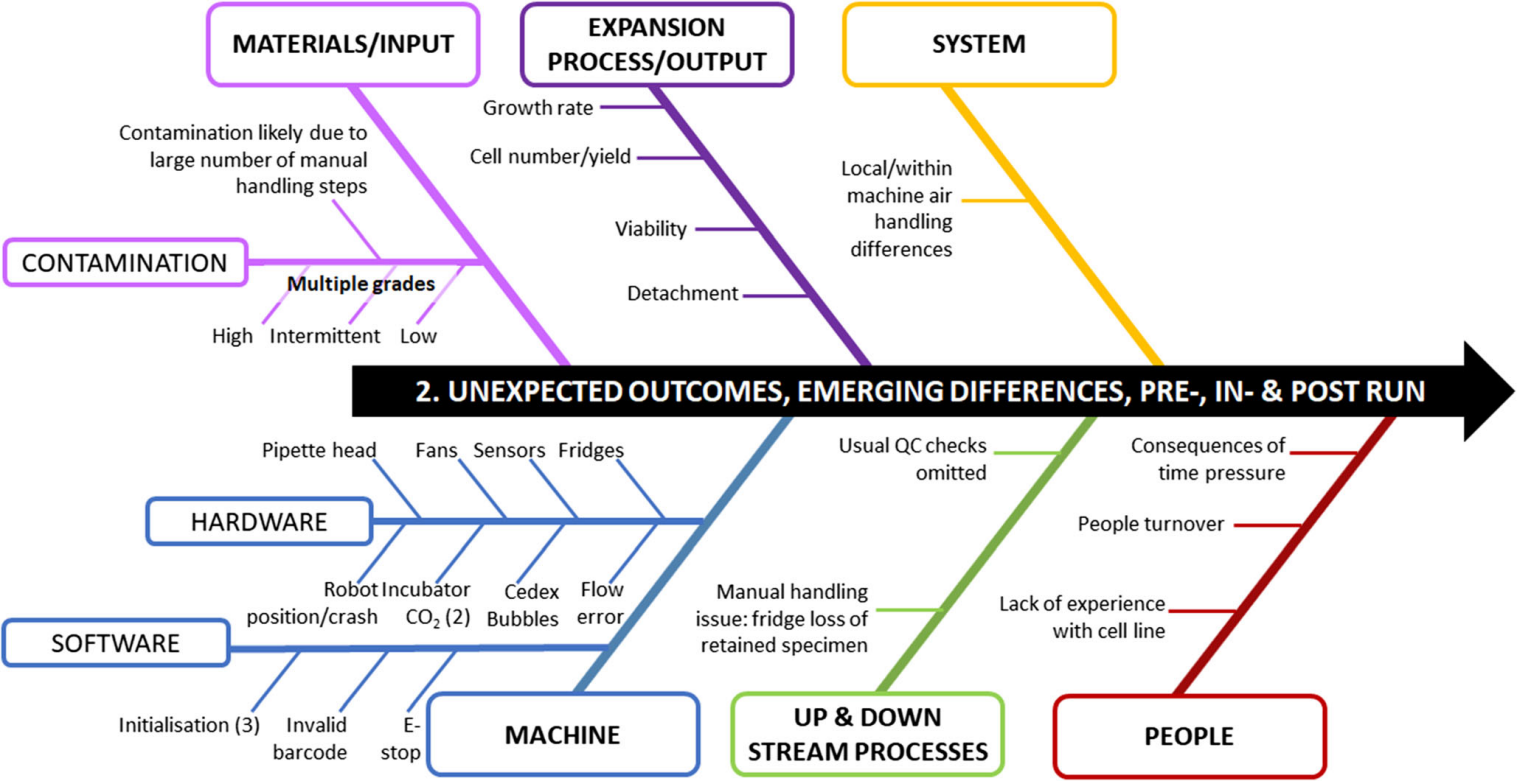
Ishikawa or fish-bone diagram illustrating the unexpected manufacturing process deviations that only became apparent following experimental runs grouped into broad areas and by individual issues

As the experiment progressed many more deviations became apparent, Fig. 6 summarises these. During the installation and operational qualification (IQ and OQ) and PQ equivalent preparatory periods including engineering runs, many software-driven deviations arose including the consequences of software and machine interactions. Whilst a number of these were due to gaps in the experience of the engineering team, some were a consequence of use the of the flexible software intensive sub-systems with redundancy—error-prone features—and of the use of custom software. These are strong signals for machine designers and programmers seeking to exploit programmable components (for instance the handling robot at the core of the machine) that permit fast machine design and create more generic process development platforms to focus on robust code design and on coding steps that may raise errors.

Given that the materials used in advanced therapeutic medicinal product (ATMP) manufacturing can be very rare—a single patient sample that forms the process starting material for instance—as discussed above it is necessary to consider whether such contingency actions should be permitted in automated production systems. Keeping in mind that such interventions maybe very difficult in closed automated systems. An example of such a contingency action encountered in this experiment is a manual intervention following a machine failure to extract a flask from the machine and to manually pipette out the contents to permit recovery of the product at the last process step. Clearly from a comparability perspective for this particular experiment, each site would have to use the same contingency actions, the machine and process is required to be returned to its desired state in the same way at all sites. The consequences of such in-process interventions, with respect to product risk must be understood and managed.

As the experiment progressed further, some significant unplanned deviations arose with the consequences of risk to the product. The most visible of these was obvious contamination, echoing industry level discussions on the prevention of microbial and virial contamination [21], with a clear requirement to scrap the product. This highlighted that the process should be closed to reduce risk, however, in the protocol employed in this work, conventional T-flasks were used. Other deviations were associated with incubator atmosphere failure. These would be preventable in a manufacturing setting via preventative maintenance and more attention to OEE.

The most challenging and difficult to manage deviation was the observation of a low-level contamination at different levels in cultures at one site, the consequences of this on growth rate are shown in Fig. 3. This echoes recent observations made in the production of preclinical trials batches [28]. In order to mitigate this deviation, it is suggested that post-process visual microscopic morphological comparison is performed, in this instance ‘post-processing’ refers to the completion of each process step, i.e. medium exchange and cell passage. Specifically, following the completion of each process step, the operator should collect a sample of excess cell suspension from each cell flask. This sample medium requires visual examination via light microscopy to determine the presence of any discrepancies. This can include media turbidity, pH deviations (as indicated by the phenol red indicator) and the presence of any floating debris.

These unexpected deviations highlighted the need for (real-time) monitoring techniques to permit early identification of incipient contamination and re-emphasised the need for agreed visual controls to permit a light microscopy and imaging approach to this, the most familiar approach to biological scientists. This capability is included in second-generation machines. The initial protocol assumed that operators would visually examine the cultures, an imaging step was subsequently added to assist communication between sites. Subsequently, this step was critical when assessing potentially contaminated cultures. This in turn identified a requirement to have consistent visual standards as part of the SOP that all parties would interpret in the same way. These visual standards are required to represent all potential states of the culture. Such standards are still required for automated imaging. Furthermore, it is recommended that in-house randomised in-depth analysis using quantitative analytical techniques including qPCR and metabolite analysis screening are performed to act as an additional quality control measure to definitively ensure that culture sterility. An additional level of process scrutiny would be the inclusion of routine external sterility testing to identify issues before and during the process.

Noting that the original master and working cell banks were mycoplasma-tested, investigation showed that contamination did not appear to be across the whole of the experimental working bank and highlighted the need to be able to non-destructively monitor individual vials. Further investigations were compromised by cold storage and courier issues but indicated that not all the cryovials used in the experiment were contaminated. The discussion that arose as a consequence of the incipient contamination and the lack of clarity on whose role it was to authorise the termination of the culture process confirmed the need for a carefully structured quality system across the sites with clear roles for local, remote and overseeing decision-makers (i.e. the equivalent of the QP in the experiment) and escalation procedures. The difficulty in establishing the original source/s of contamination highlights the need for strict, comprehensive quality control of the master banks; followed by continuous qualification of the working banks. Furthermore, there is a requirement to monitor the condition of vials as they travel through the supply chain.

The nature of ‘unexpected deviations’ and how they are dealt with highlights the requirement for a quality system that will change with time, which emphasises the importance of change management protocols across sites. Consideration of candidate corrective and preventative actions for the low-level contamination explored the following: (i) the delivery of the product to the patient with antibiotics (recently discussed at Phacilitate [24]) is under the guidance of the treating physician; however, this may pose the risk of litigation as has recently occurred in the USA [11] following a regulatory recall [22]. This approach also does not address viral contamination. (ii) The culture of the product with antibiotics; this, however, may mask other issues within the culture and again does not address issues of viral contamination. (iii) The qualification of the starting bank to agreed enhanced standards. (vi) The improvement of the current process by taking the steps of process closure, ensuring that all processes within the whole of the overall materials handling and storage chain do not allow contamination, and the development of agreed visual controls and other, contamination or risk-specific, check or routine monitoring techniques (i.e. sterility testing to identify issues).

**Fig. 7:**
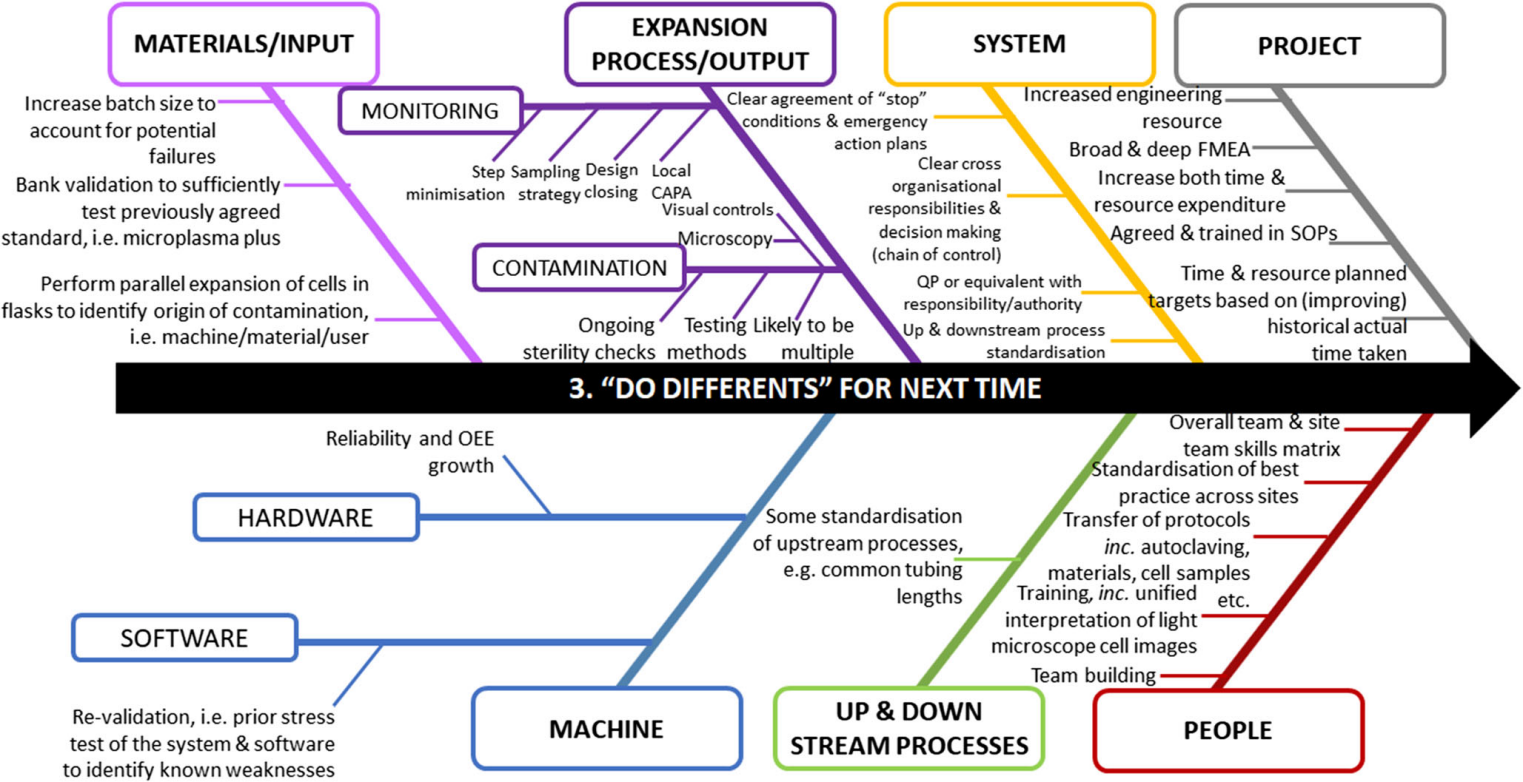
Ishikawa or fish-bone diagram illustrating post project learning or ‘do differents’ to be considered for future manufacturing scenarios, grouped into broad areas and by individual issues

Further discussion on monitoring raised the consideration that, for every process step completed by the machine, there should be an additional check step within the machine; this could be achieved via the machine itself or an additional internal process within the machine system and that the results of the check should form part of the batch record. Ensuring the quality of open manual interventions must also be considered, for instance using a two-person ‘buddy’ system to view and check all actions. An instance of this is the use of a checker to check, view and confirm compliance to the SOP in addition to updating manufacturing batch records in real time [8, 12], especially given that the QP is reliant upon the batch record to release the product. The financial consequences of these issues are significant as is the requirement, in the multiple site settings, for a local quality assurance organisation in which in the QP can be confident. Figure 7 summarises these ‘do differents’ and other learning points from the project that form key areas for further work.

### Contamination

This experiment has emphasised the issues associated with contamination, especially low-level, uncharacteristic contamination, and how this should be managed in a multiple sites setting within a European regulatory context. The term ‘uncharacteristic’ refers to the fact that during this experiment, whilst there were contaminations in cell cultures, there was no medium clouding or turbidity observed in any of the cultures. These are normally seen in many cultures with bacterial contamination [23].

To address this, the first step is the establishment of a ‘central’ QP role and the identification and recruitment of a QP sensitive to the issues of manufacturing at multiple sites. The QP is required to determine operator roles and requirements, this is inclusive of specific training requirements based on a defined set of competencies. The role of the QP must be agreed across all sites since they will assume ultimate responsibility for the manufacturing process. Similarly, the roles and expectations of all operators across all sites must to be defined, agreed and formalised contractually. This contract is an agreement by all operators to ensure that no deviations in timings, process parameters or incidents due to personal circumstances may occur.

In situations where operator roles may deviate, for example due to illness, relocation, staff turnover or change of employment, the QP should to be notified with as much notice as possible. For clinical trials or marked production, given their responsibility for product release, the QP should have the final authority on reassignment of operator roles and responsibilities and whether operators are appropriately qualified. This should not be the decision of the individual sites, but the QP should consider the input of individual site operators and local quality personnel. Similarly, any decisions with regard to how to manage contamination must ultimately be decided by the QP, using all recorded data. The QP should be able to request additional testing if required.

This experiment has highlighted the importance of imaging data. In the experiment described here, individual operators had, or were, assuming the responsibility of making decisions with regard to continuation of manufacturing when contamination occurred, for example, when only one flask was showing signs of contamination; this responsibility should be removed from the individual. Ideally such decisions should be made by an on-call QP, not the operator. However, it is acknowledged that this may not be feasible in all instances, thus there is a requirement for a quality hierarchy; it is recommended that there is an additional QA resource that can act as an auxiliary to the QP at every site with carefully designed responsibility who reports in a timely way to a QP at a centralised location.

The clear ultimate responsibility of the QP for any decisions regarding the manufacturing process, training and competency requirements, and Corrective Action Preventive Action (CAPA) should help avoid stigma associated with contamination or process failures especially when addressed within a quality improvement framework. There should be an agreed acceptance across all operators and sites that contamination is an inevitable risk of cell manufacturing and should be reported immediately to the QP without questioning of the operator’s cell culture practice given that there are many sources for contamination. A single database is required for the recording of contamination or process failures across all sites. A standardised format of reporting and paperwork would allow the QP to identify and analyse general trends and common areas of error or contamination.

Prior to the onset of any extended processing, a detailed screening and re-qualification of banks is recommended; this should occur in a single centralised unit to avoid deviations in protocols and analysis. This process could also be blinded to avoid any potential bias in analysis and teams involved should include manufacturing and quality representatives of each site involved. All operator training needs should be reviewed and signed off centrally by the QP or their representative. Experimentation and extended processing are only to commence upon completion and sign-off of operator training records. The training documentation is a record that each operator has demonstrated and completed all the required competencies required to work with the defined cell line and protocols associated. Training is required for all involved in the cell manufacturing process, irrespective of their role; this is to be complemented by peer-to-peer, buddy, observation and note-taking. All aspects from manual handling to automation need to be covered during the training procedure; progression to automated culture can only occur once manual culture competencies has been signed off by the QP.

It is vital that all prior training is specific to the cell line being manufactured, the process protocol and production system, and the product; a one-size fits all approach is not appropriate. In order to ensure and determine that cell specific parameters such as cell growth rate and viability are recognised by all operators and across all sites, pre-run experiments should be designed. This will enable a consensus regarding a cell line–specific standard range of acceptable growth rates, viability and phenotype expression. This strategy circumvents any biological discrepancies and deviation during actual process runs since any deviations and potential errors that fall outside of the agreed operation window process limits will be identified and recorded during training and process development. Additionally, a mathematical modelling approach can be applied to predict the growth rate and dynamics of specific cell lines and determine the critical process parameters (CPPs) that affect the metabolite concentration, viability and growth. Such parameters include cell density, composition of bulk media, presence and concentration of growth factors, the timing and frequency of feeding. This approach can also be used in predicting the effect of variation in temperature and incubation time. The results of this approach can be recorded in a centralised database to permit the establishment of agreed limits for biological process deviation across sites.

During the product/process development and training processes, all operators should record and report potential areas for error, process deviation and risk. These are to be reported using a standardised format on a centralised database to be viewed by the QP. Ultimately a single, detailed SOP that includes the risk mitigations identified should be prepared. The use of decision-process flowcharts for the SOP should be considered for steps of the process where conditional actions are acceptable. This should address the entire manufacturing process, and the conditions for terminating the culture process made explicit. Accompanying the process SOP, there is a requirement for a harmonised and uniform SOP for machine and equipment maintenance, synchronised to usage and workload. This will serve as a means to reduce points of failure regarding equipment and machine malfunction, whilst also ensuring that all processing tools are serviced, calibrated and equivalent across sites.

This experiment was carried out by a collaboration of manufacturing aware development organisations with strong translational missions. Despite these common goals, each organisation had different perspectives and cultures. In retrospect, each institute should have run an in-depth risk assessment of potential process and equipment deviations and any potential sources of and areas of risk for contamination. It would have been particularly helpful if the in-depth risk assessment also involved the wider laboratory teams. This should have been followed by sharing and discussion of proposals for mitigation by the three sites together and these, when agreed by all organisations and approved by the QP equivalent, included within the SOP prior to the onset of any work commencing. Once agreed, no deviations to the SOP are permitted unless they are progressed through appropriate change control.

## Conclusions

Establishing how to effectively manufacture cell therapies is an industry level problem. Decentralised manufacturing is of increasing importance and its challenges are recognised by healthcare regulators with deviations receiving specific attention from them. This paper is the first to report the deviations and other risks encountered when implementing comparability of expansion of human pluripotent stem cells in an automated three international site–decentralised manufacturing setting. In addition to identifying practically encountered process deviations and how they may be addressed the work has identified incipient process failures including low-level contamination that are intrinsically difficult to manage and exacerbated by a decentralised process. In summary, it has highlighted the following issues:

- The practical difficulties associated with the choice of a representative and stable cell line to be used as a manufacturing standard.
- All sites involved are required to contribute to a single agreed SOP to permit learning from all sites to be considered and ensure that all sites are aligned behind the protocol. Expertise and training differences between sites must be addressed to ensure all sites are competent.
- Compromises may be required between sites to minimise variations due to different work patterns and differences in legal and regulatory frameworks.
- Resolving differences between sites pre- and post-preventative maintenance required significant supplier interaction; therefore, supplier capacity issues need to be considered as part of a realistic project planning. Differences between sites may reduce the practical allowable process window.

Differences and deviations are particularly apparent in a multiple site setting, deviations identified in this work and the actions required to address them include:

- Identification of equipment failures and the need for recommissioning.
- Timely interventions for process recovery.
- The consequences of use of flexible software intensive sub-systems.
- Differences in cell culture process output.
- Agreed visual controls via light microscopy machine imaging written into the protocol.
- Consistent representative visual standards as part of the SOP that all parties would interpret in the same way. These visual standards are required to represent all potential states of the culture.
- Correction of deviations in the same way at all sites—and critically agreement of the cause across sites.
- Common quality system including QP structure capable of evolution with time.
- Manual processes require the use of check steps and operator buddies, these have significant financial implications.
- In process sterility monitoring should be present, but may impact product yields due to the significant requirements for sampling.

This experiment has confirmed where key components and additional costs of GMP are added to the basic costs of the cell culture manufacturing process, and particularly show multiple sites production requires additional QA resource. Automation is an obvious way forward in the manufacturing of ATMPs because of its scalability, but it is necessary to be clear about the problems to be solved for it to be unquestionably applied as an alternative to manual processing. This paper has especially emphasised the requirement for monitoring steps to be included within the machine system.

As a final comment, it should be noted that the level of effort required to execute this project was significantly underestimated by the project partners. The level of budget required to completely validate a distributed manufacturing multiple sites manufacturing strategy would only be justified in commercial settings where the financial return is significant. Further work is still required however to better understand how to design a compliant multiple site process validation approach that is cost effective.
